# CXCR2 is a negative regulator of p21 in p53-dependent and independent manner via Akt-mediated Mdm2 in ovarian cancer

**DOI:** 10.18632/oncotarget.24231

**Published:** 2018-01-15

**Authors:** Rosa Mistica C. Ignacio, Yuan-Lin Dong, Syeda M. Kabir, Hyeongjwa Choi, Eun-Sook Lee, Andrew J. Wilson, Alicia Beeghly-Fadiel, Margaret M. Whalen, Deok-Soo Son

**Affiliations:** ^1^ Department of Biochemistry and Cancer Biology, Meharry Medical College, Nashville, TN 37208, USA; ^2^ Department of Pharmaceutical Sciences, College of Pharmacy, Florida A&M University, Tallahassee, FL 32301, USA; ^3^ Department of Obstetrics and Gynecology, Vanderbilt University Medical Center, Nashville, TN 37232, USA; ^4^ Vanderbilt-Ingram Cancer Center, Vanderbilt University Medical Center, Nashville, TN 37203, USA; ^5^ Division of Epidemiology, Department of Medicine, Vanderbilt University Medical Center, Nashville, TN 37203, USA; ^6^ Department of Chemistry, Tennessee State University, Nashville, TN 37209, USA

**Keywords:** Akt, CXCR2, Mdm2, ovarian cancer, p53

## Abstract

Ovarian cancer (OC) has the highest rate of mortality among gynecological malignancy. Chemokine receptor CXCR2 in OC is associated with poor outcomes. However, the mechanisms by which CXCR2 regulates OC proliferation remain poorly understood. We generated CXCR2-positive cells from parental p53 wild-type (WT), mutant and null OC cells, and assessed the roles of CXCR2 on proliferation of OC cells in p53-dependent and independent manner. CXCR2 promoted cell growth rate: p53WT > mutant = null cells. Nutlin-3, a p53 stabilizer, inhibited cell proliferation in p53WT cells, but had little effect in p53-mutant or null cells, indicating p53-dependence of CXCR2-mediated proliferation. CXCR2 decreased p53 protein, a regulator of p21, and downregulated p21 promoter activity only in p53WT cells. The p53 responsive element (RE) of p21 promoter played a critical role in this CXCR2-mediated p21 downregulation. Moreover, CXCR2-positive cells activated more Akt than CXCR2-negative cells followed by enhanced murine double minute (Mdm2). Silencing Mdm2 or Akt1 upregulated p21 expression, whereas Akt1 overexpression downregulated p21 at the promoter and protein levels in p53WT cells. Cell cycle analysis revealed that CXCR2 decreased p21 gene in p53-null cells. Interestingly, romidepsin (histone deacetylase inhibitor)-induced p21 upregulation did not involve the p53 RE in the p21 promoter in p53-null cells. Romidepsin decreased the protein levels of Akt1 and Mdm2, leading to induction of p21 in p53-null cells. CXCR2 reduced romidepsin-induced p21 upregulation by activating Akt-induced Mdm2. Taken together, CXCR2 enhances cell proliferation by suppressing p21 through Akt-Mdm2 signaling in p53-dependent and independent manner.

## INTRODUCTION

Ovarian cancer is the deadliest gynecological cancer and the fifth leading cause of cancer-related deaths in women in the U.S [1]. Despite the heterogeneity on malignancies, ovarian cancer is simplified into two subtypes, type I and II tumors [2, 3]. Type I tumors include low-grade, mucinous, endometrioid and clear cells, in which the lesion is fully characterized, leading to a better prognosis. On the other hand, type II tumors consist of high-grade serous and undifferentiated cells, wherein the lesion is not fully identified and is quite widespread, resulting in aggressiveness with a poor prognosis [2, 4, 5]. In addition, type I tumors have genetic feature such as lack of mutation of p53, while type II tumors have high frequency of p53 mutation [2, 6].

Cumulative studies indicate that ovarian cancer is associated with chronic inflammation [7, 8]. Proinflammatory chemokines are now recognized as critical mediators in the tumor microenvironment and their amplified expression parallels to tumor growth, angiogenesis, and metastasis in many human cancers [9-13]. CXC chemokines such as CXCL1, CXCL2, CXCL3, CXCL5, CXCL6 and CXCL8 induce potent angiogenesis mediated by the G-protein-coupled receptor CXCR2 [14, 15]. Increasing evidence has implicated a high expression of CXCR2 in advanced stage of ovarian carcinomas leading to increased cancer progression and enhanced angiogenesis [16]. High expression of CXCR2 or its ligand CXCL1 leads to increased ovarian cancer proliferation, which is believed to be partly mediated by transactivation of EGFR signaling, and the suppression of CXCR2 leads to apoptosis [16, 17]. In addition to clear evidence of a role for CXCR2 in cancer progression, CXCR2 is known to influence oncogene-induced and replicative senescence through a DNA-damage [18]. The molecular mechanism of CXCR2 in cancer progression may include various signaling pathways such as mitogen-activated protein kinase (MAPK), phosphoinositide 3-kinase (PI3K), signal transducer and activator of transcription 3 (STAT3) and NF-κB pathways [16]. Our previous study confirmed the role of NF-κB signaling through EGFR-transactivated Akt on the CXCR2-driven ovarian cancer progression [19, 20]. Although CXCR2 regulates cell cycle through multiple signaling pathways, the mechanistic effects of CXCR2 on ovarian cancer cell proliferation is poorly understood.

Despite advanced progress on the roles of the p53 in regulating cell cycle and proliferation, little is known about how CXCR2 contributes to the proliferation of ovarian cancer in p53-dependent and independent manner. This study was designed to investigate the molecular mechanisms of CXCR2-mediated proliferation in p53 wild-type (WT), mutant and null ovarian cancer cells.

## RESULTS

### CXCR2 promotes cell proliferation in ovarian cancer cells

**Figure 1:**
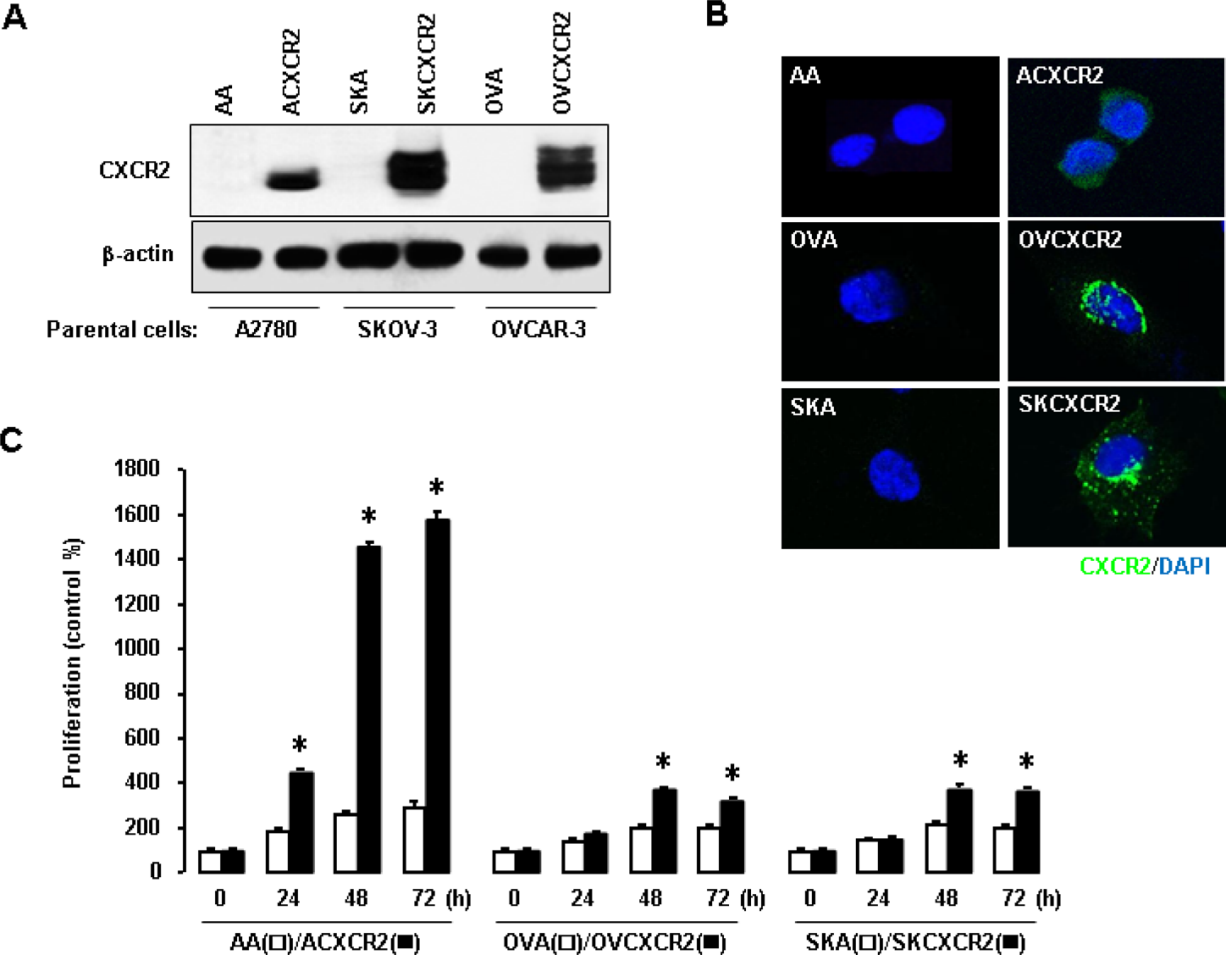
Enhanced effects of CXCR2 on cell proliferation in ovarian cancer cells (**A–B**) Confirmation of CXCR2 protein expression in CXCR2 stably transfected ovarian cancer cells A2780 (ACXCR2), OVCAR-3 (OVCXCR2) and SKOV-3 (SKCXCR2) versus their negative CXCR2 counterparts (AA, OVA, SKA), respectively, by western blot and confocal imaging analysis. β-actin was detected as an internal loading control of cell lysates. A representative result is shown from duplicated experiments. (**C**) CXCR2 promotes increase cellular proliferation in CXCR2-positive cells compared to its negative cells. All data are shown as mean ± SE from triplicated experiments. ^*^ indicates a significant increase (*p* ≤ 0.05) by Student’s *t*-test at appointed time points.

Based on representative human ovarian cancer cell lines, A2780 (p53WT, [21]), OVCAR-3 (p53-mutant R248Q, [22, 23]) and SKOV-3 (p53-null, [22, 24]), we generated CXCR2-negative (AA, OVA, SKA) and positive (ACXCR2, OVCXCR2, SKCXCR2) cells via stable transfection with empty vector and CXCR2 vector, respectively. Western blotting and confocal imaging analysis confirmed the relative expression levels of CXCR2 in the generated cell lines (Figures 1A and 1B). Thereafter, we compared cellular proliferation in CXCR2-positive vs. negative cells. The proliferation was assessed at 24, 48 and 72 h. CXCR2-positive ovarian cancer cells significantly increased in proliferation compared to negative cells (Figure 1C). ACXCR2 cells had a higher proliferative rate than OVCXCR2 and SKCXCR2 cells (Figure 1C), indicating negative effects of CXCR2 on p53-suppressed cell proliferation.

### Nutlin-3 dramatically reduces proliferation of p53WT cells in which CXCR2 inhibits p21 expression

**Figure 2:**
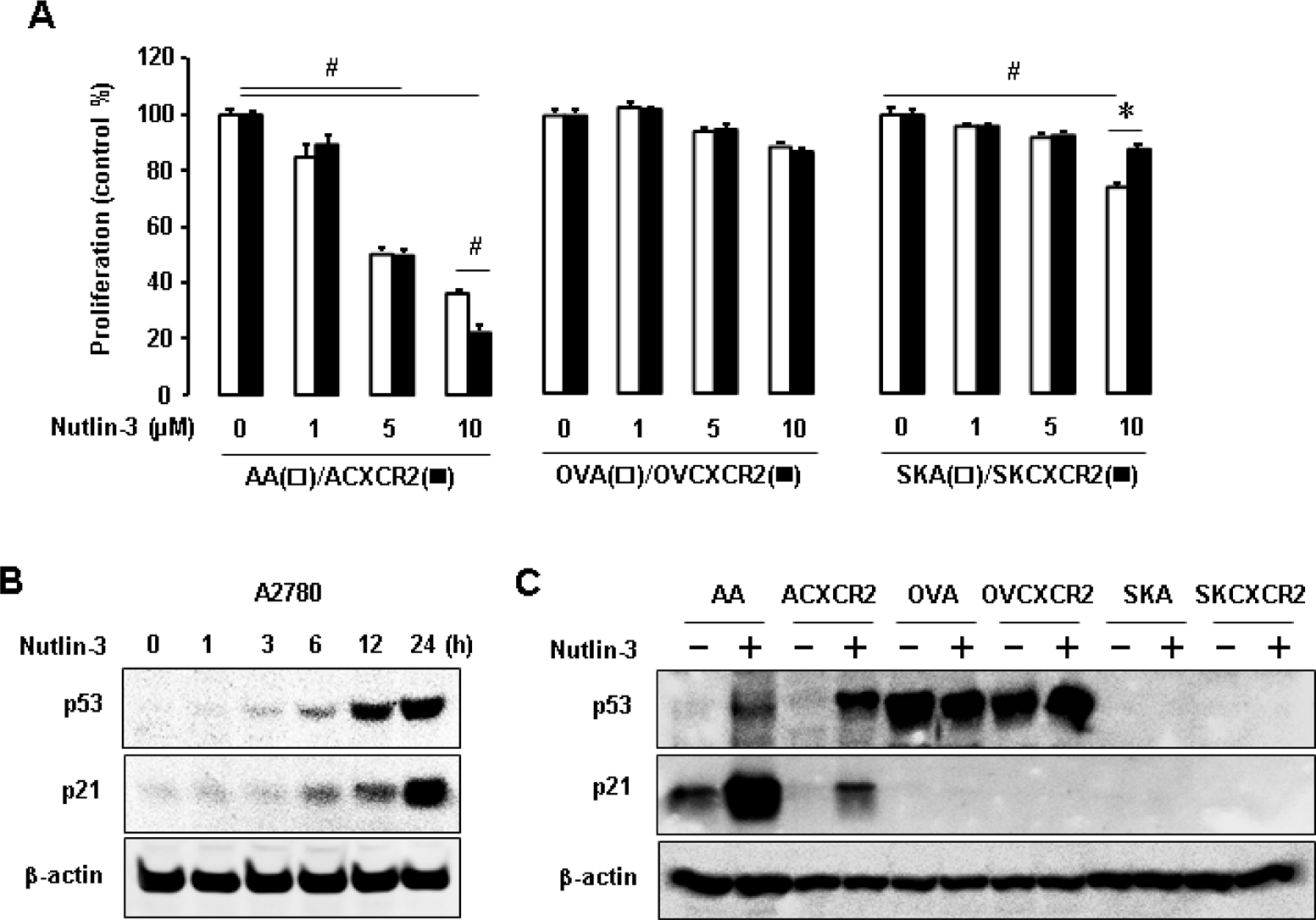
The p53-dependent effects of nutlin-3 on cell proliferation and p21 levels in p53WT, mutant and null cells (**A**) The p53-dependent effect of nutlin-3 on cell proliferation in CXCR2-negative vs. positive cells with different p53 status. All data are shown as mean ± SE from triplicated experiments. ^#^ and ^*^ indicate a significant decrease and increase (*p* ≤ 0.05) by ANOVA and Student’s *t*-test, respectively. (**B**) Time-dependent effect of nutlin-3 on p53 and its downstream p21 expression in p53WT A2780 cells. (**C**) Comparative induction of nutlin-3 on p53 and p21 protein levels in p53WT cells (AA/ACXCR2), p53-mutant (OVA/OVCXCR2) and p53-null (SKA/SKCXCR2) cells. β-actin was detected as an internal loading control of cell lysates. A representative result is shown from duplicated experiments.

We employed nutlin-3, a known inhibitor of p53-Mdm2 (murine double minute) complex, to define inhibitory effects of CXCR2 on p53-dependent signaling. Nutlin-3 significantly inhibited cellular proliferation in p53WT AA and ACXCR2 cells in a dose-dependent manner (Figure 2A). At 10 µM of nutlin-3, ACXCR2 cells were more inhibited than AA cells, indicating sensitive response of CXCR2 on p53-dependent proliferation in p53WT cells. However, p53-mutant OVA/OVCXCR2 and p53-null SKA/SKCXCR2 cells had a decreased trend on proliferation, but no significant change with nutlin-3 treatment (Figure 2A). Moreover, SKA cells were more inhibited after treatment with nutlin-3 at high dosage (10 µM) compared to SKCXCR2 cells (Figure 2A), indicating the resistant response of CXCR2 on nutlin-3-mediated inhibition of cell proliferation in p53-null cells. We then assessed if nutlin-3 could affect the protein levels of p53 and its transcriptional target p21. Nutlin-3 upregulated p53 and p21 levels in a time-dependent manner in p53WT A2780 cells (Figure 2B). Nutlin-3 blocked p53 degradation followed by increased p21 levels in p53WT (AA/ACXCR2) cells, but not in p53-mutant (OVA/OVCXCR2) or p53-null (SKA/SKCXCR2) cells (Figure 2C). CXCR2 reduced nutlin-3-mediated increase of p21 protein levels (Figure 2C). These results suggest negative effects of CXCR2 on the p53-dependent p21 regulation of nutlin-3 only in p53WT cells.

### CXCR2 reduces p21 promoter activity in p53WT cells

**Figure 3:**
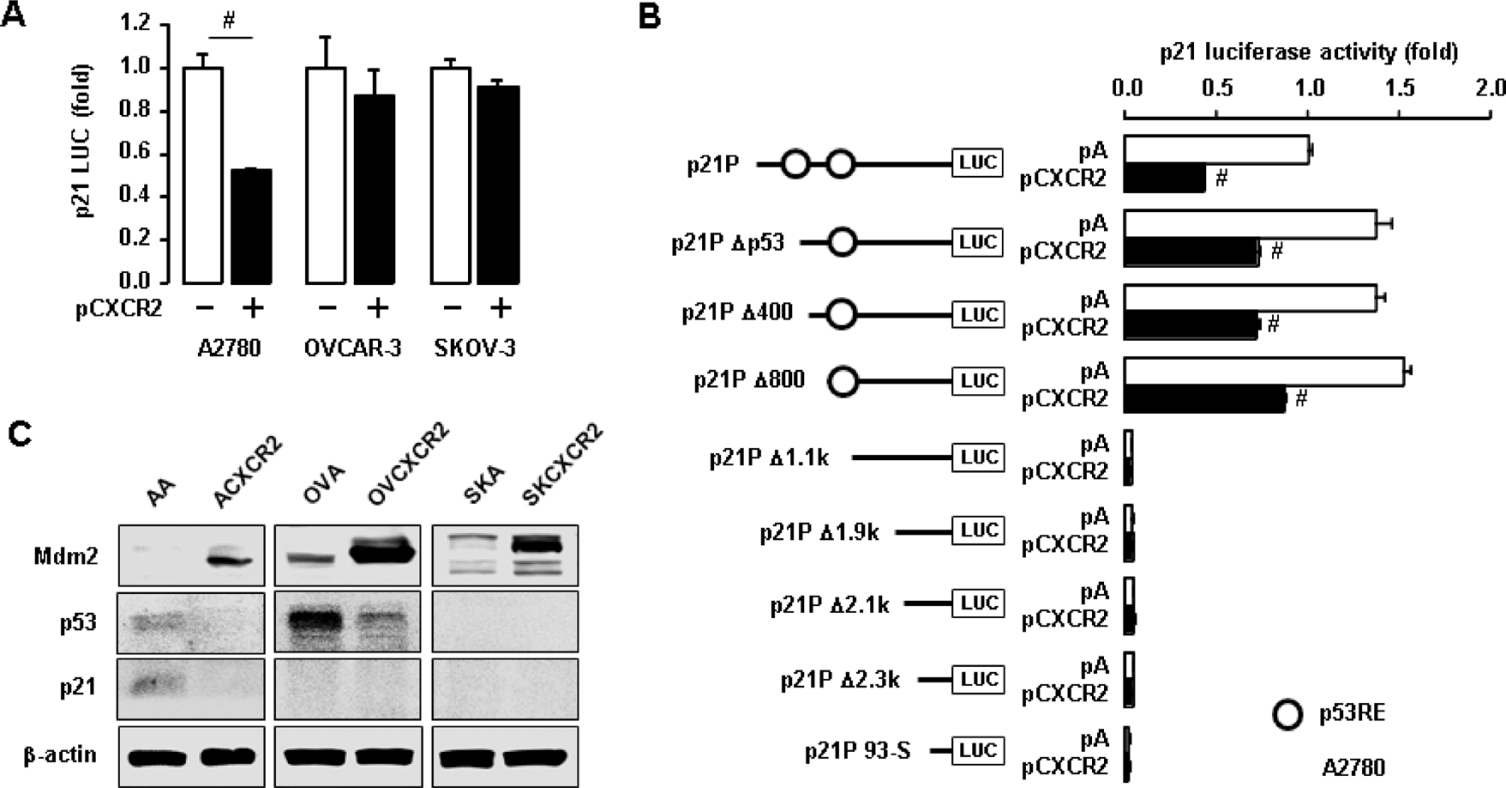
Negative effects of CXCR2 on p21 promoter activity in a p53-dependent manner (**A**) Differential effects of CXCR2 on p21 promoter activity in p53WT A2780 cells, p53-mutant OVCAR-3 and p53-null SKOV-3 cells. (**B**) The p53-dependent inhibition of CXCR2 on p21 promoter activity in deleted constructs of p21 promoter in p53WT A2780 cells. All data are shown as mean ± SE from triplicated experiments. (**C**) Induced effects of CXCR2 on Mdm2 protein in p53-dependent p21 regulation. β-actin was detected as an internal loading control of cell lysates. A representative result is shown from duplicated experiments. ^#^ indicates a significant increase (*p* ≤ 0.05) in each pair by Student’s *t*-test.

We then checked if CXCR2 could affect the p21 promoter activity in ovarian cancer cells with different p53 status. Transient transfection of CXCR2 reduced p21 promoter activity in p53WT A2780 cells, but had no effects in p53-mutant OVCAR-3 or p53-null SKOV-3 cells (Figure 3A). To identify critical promoter regions to regulate p21, we determined the effect of CXCR2 on p21 luciferase activity in deleted constructs of p21 promoter [25]. The p53 responsive element (RE) (-1395/-1376) between p21P Δ800 and p21P Δ1.1k constructs played a critical role in CXCR2-mediated p21 downregulation in a p53-dependent manner (Figure 3B). Additionally, we checked the expression level of Mdm2, a negative regulator of p53 and an E3 ubiquitin ligase, in CXCR2-negative and positive cells. CXCR2 upregulated Mdm2 protein levels followed by decreased p53 and p21 protein levels in p53WT cells (Figure 3C). The p53-mutant and null cells also showed induced Mdm2 protein levels in response to CXCR2 (Figure 3C). Interestingly, CXCR2-mediated Mdm2 induction downregulated p53 protein levels in p53-mutant cells (Figure 3C), despite the nonfunctional role of p53 mutant protein compared to p53WT protein.

### CXCR2 activates Akt followed by induced Mdm2 protein to downregulate p53-dependent p21 expression in p53WT cells

**Figure 4:**
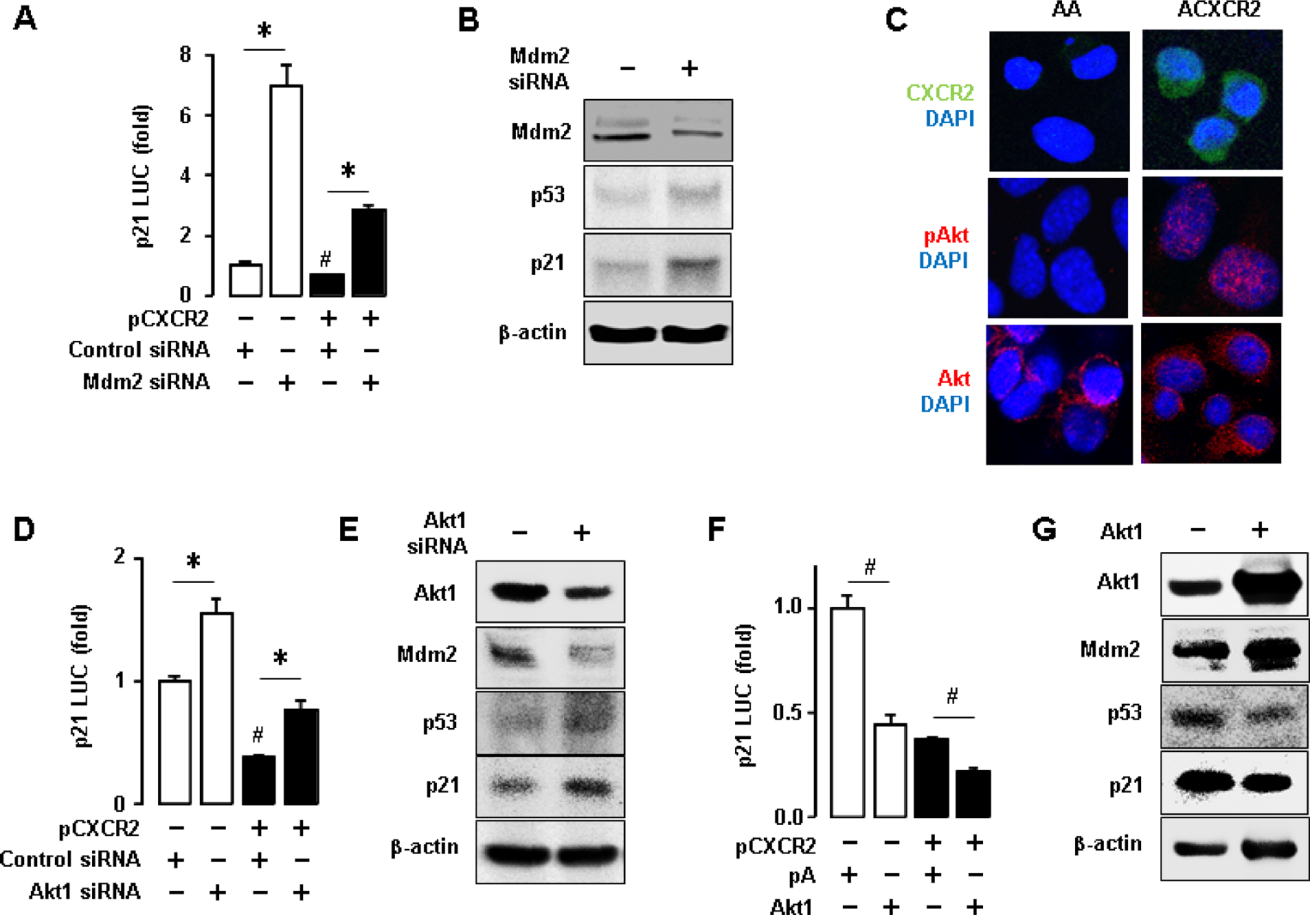
Inhibitory effects of CXCR2-derived Akt-Mdm2 axis on p53-dependent p21 protein expression in p53WT cells (**A**) Knockdown of Mdm2 increased p21 promoter activity and recovered the CXCR2-mediated downregulation of p21. (**B**) Confirmation of the knockdown of Mdm2 protein through western blot and the effect of knockdown of Mdm2 protein on p53-dependent p21 protein expression. (**C**) Comparison of Akt activation in AA and ACXCR2 cells by confocal imaging analysis. (**D**) Knockdown of Akt1 increased p21 luciferase activity and recovered the CXCR2-mediated downregulation of p21. (**E**) Confirmation of the knockdown of Akt1 through western blot and the effect of knockdown of Akt1 on Mdm2, p53 and p21 protein expression levels. (**F**) Overexpression of Akt1 decreased the p21 luciferase activity and further abated the CXCR2-mediated downregulation of p21. (**G**) Confirmation of the overexpression of Akt1 through western blotting and the effect of silencing Akt1 on Mdm2, p53 and p21 protein expression. β-actin was detected as an internal loading control of cell lysates. All data are shown as mean ± SE from triplicated experiments (A, D, F) and a representative result is shown from duplicated experiments (B, C, E, G). ^*^ and ^#^ indicate a significant increase and decrease (*p* ≤ 0.05), respectively, by Student’s *t*-test.

To further assess the involvement of Mdm2 in CXCR2-mediated p21 downregulation, we utilized short interfering RNA (siRNA) to knockdown Mdm2 protein. Knockdown of Mdm2 induced p21 promoter activity and partially restored CXCR2-mediated inhibition of p21 promoter activity in p53WT A2780 cells (Figure 4A). We verified that knockdown of Mdm2 upregulated the p53 and p21 protein levels (Figure 4B). It is well established that Akt induces Mdm2 [26]. Thus, we checked the expression level of Akt in CXCR2-negative or positive cells using confocal imaging. ACXCR2 cells expressed more activated Akt than AA cells (Figure 4C). Among the Akt isoforms, Akt1 was frequently elevated in ovarian cancer [27], and our representative ovarian cancer cell lines showed higher protein expression of Akt1 [28]. Further, we made knockdown of Akt1 using siRNA to check the effects of Akt on p21 promoter luciferase activity. Knockdown of Akt1 significantly upregulated p21 promoter activity, and partially restored the CXCR2-mediated downregulation of p21 promoter activity (Figure 4D). Protein expression data validated this result that knockdown of Akt1 downregulated Mdm2 protein levels followed by the p53-dependent p21 upregulation (Figure 4E). The transient transfection of Akt1 expression vector in A2780 cells led to the reduced p21 promoter activity and further caused the CXCR2-mediated p21 downregulation (Figure 4F). We validated that Akt1 overexpression upregulated Mdm2 protein followed by downregulation of the p53-dependent p21 protein expression (Figure 4G). These results indicate that CXCR2 suppresses cell cycle inhibitor p21 protein, probably by downregulating p53 via Akt-Mdm2 axis.

### CXCR2 negatively regulates cell cycle inhibitor p21 in p53-null ovarian cancer cells

**Figure 5:**
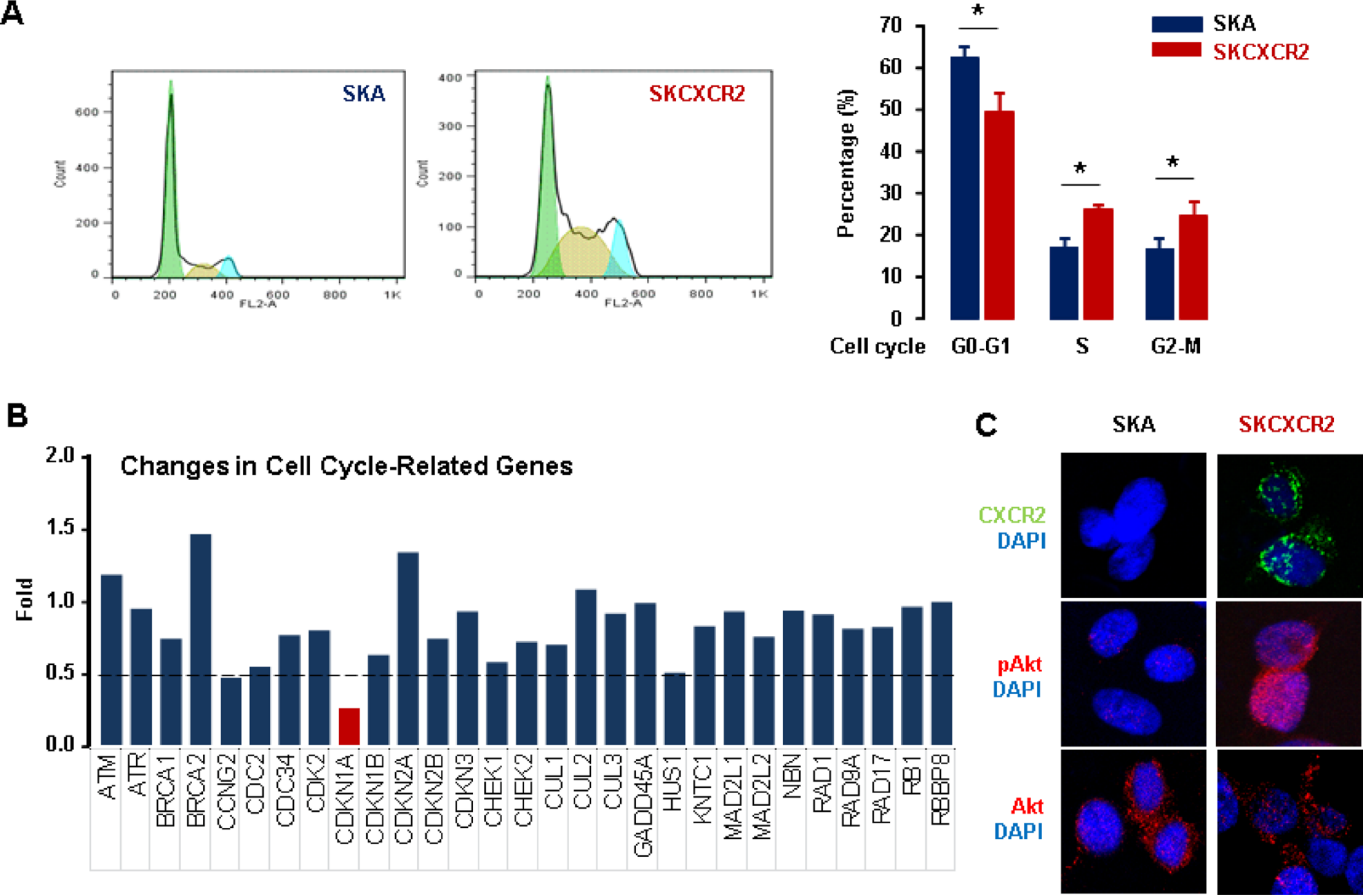
Differential effects of CXCR2 on cell cycle between p53-null SKA and SKCXCR2 ovarian cancer cells (**A**) Comparison of cell cycle between SKA and SKCXCR2 cells by flow cytometry. All data are shown as mean ± SE from triplicated experiments. ^*^indicates a statistical significance (*p* ≤ 0.05) by Student’s *t*-test. (**B**) Comparison of cell cycle related genes between SKA and SKCXCR2 cells by PCR array. The dotted line indicates a 0.5-fold increase; those with a <0.5-fold increase and average cycle threshold >30 are recognized as down-regulated genes. All data are shown as average from duplicated experiments. (**C**) Comparison of Akt status between SKA and SKCXCR2 cells by confocal imaging. A representative result is shown from duplicated experiments.

We demonstrated that CXCR2 suppressed p53-dependent p21 upregulation in p53WT cells. However, we wondered if CXCR2 could negatively regulate p21 in p53-independent manner in p53-null ovarian cancer cells. CXCR2 has been implicated in cell cycle and apoptosis via several molecular pathways in ovarian cancer [16]. Because CXCR2 accelerated cell proliferation in p53-null SKCXCR2 cells (Figure 1C), we assessed if there could be any changes in cell cycle between SKA and SKCXCR2 cells. First, we determined the differential effects of CXCR2 on cell cycle in SKA and SKCXCR2 cells based on parental p53-null SKOV-3 cells. Flow cytometry analysis revealed that SKCXCR2 cells had a significant decrease in G0-G1 phase and increase in S and G2-M phases compared to SKA cells (Figure 5A). In addition, we compared PCR array for cell cycle-related regulatory genes between SKA and SKCXCR2 cells. Cyclin-dependent kinase inhibitor 1 (CDKN1A, p21) mRNA was maximally down-regulated in SKCXCR2 cells compared to SKA cells (Figure 5B), despite no expression of p21 protein in p53-null cells (Figure 2C). Since Akt signaling was involved in CXCR2-mediated p21 downregulation in p53WT cells, we investigated whether CXCR2 could regulate p21 in p53-null cells in a similar way. Akt signaling is well known to regulate cell proliferation and survival [29–31]. Thus, we assessed Akt status between p53-null SKA and SKCXCR2 cells. Confocal imaging analysis showed more activated Akt in SKCXCR2 compared to SKA cells (Figure 5C). The CXCR2-mediated downregulation of p21 might be related to uncontrolled proliferation of p53-null cells, plausibly involving constitutively activated Akt.

### Romidepsin does not involve specific p53 RE of p21 promoter to regulate p21 in p53-null cells

**Figure 6:**
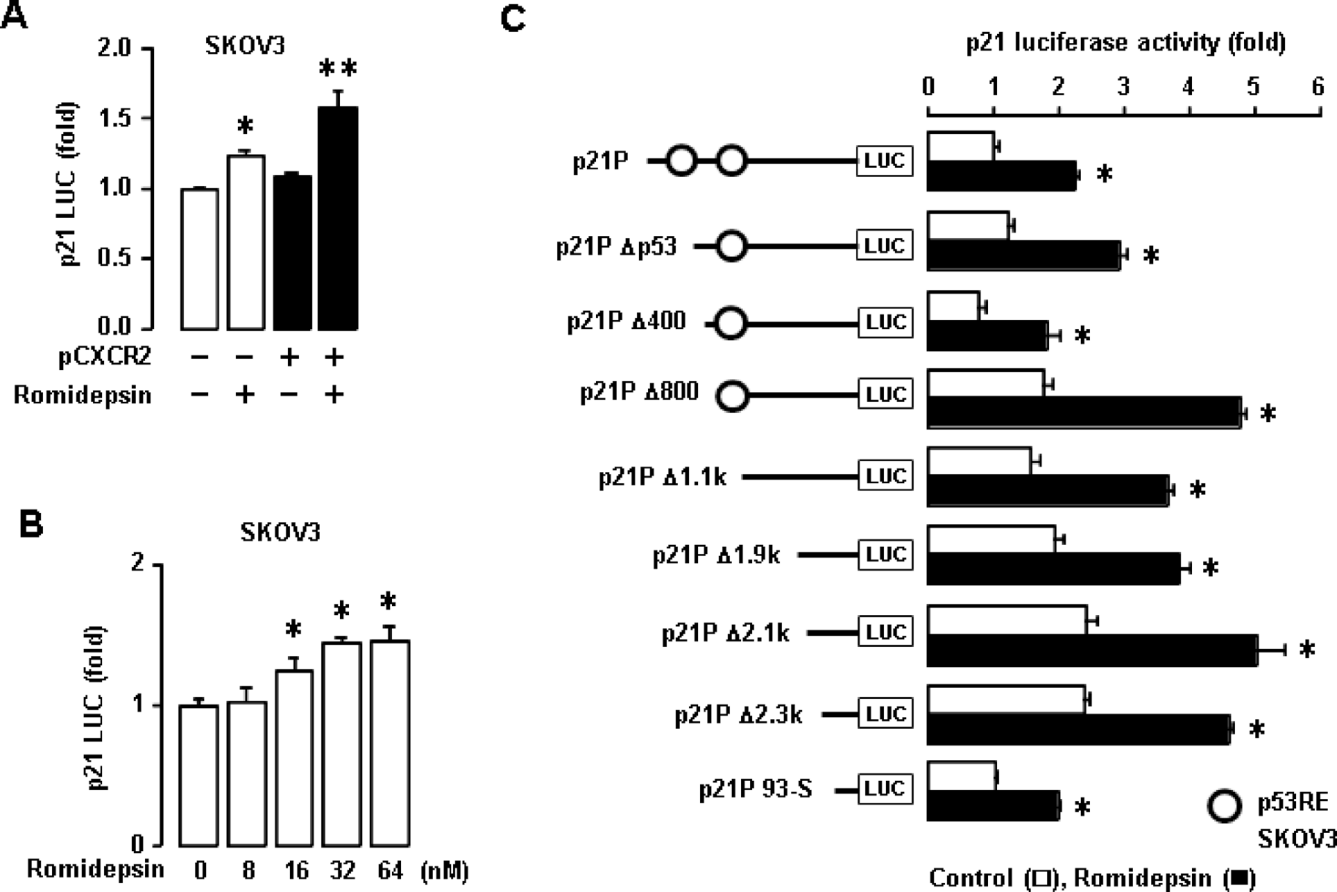
Effects of romidepsin on p21 promoter activity in p53-null SKOV-3 cells (**A**) Effects of CXCR2 on romidepsin-induced p21 promoter activity in SKOV-3 cells. (**B**) Dose-dependent effects of romidepsin on p21 luciferase activity in SKOV-3 cells. Experiments were performed in triplicate and all data are shown as mean ± S.E. ^*^(*p* ≤ 0.05) in each group by ANOVA and Tukey’s pairwise comparisons. (**C**) Effects of romidepsin on p21 promoter activity in deleted constructs of p21 promoter p53 response element in p53-null SKOV-3 cells. All data are shown as mean ± SE from triplicated experiments. ^*^indicates a statistical significance (*p* ≤ 0.05) by Student’s *t*-test.

CXCR2 had no effects on p21 promoter activity in p53-null SKOV-3 cells (Figure 3A). Furthermore, p53-null SKA and SKCXCR2 cells did not express p21 protein at basal levels (Figure 3C) despite CXCR2-mediated downregulation of p21 mRNA in p53-null cells (Figure 5B). Romidepsin (FK228), a histone deacetylase inhibitor (HDACi), is known to induce p21 protein specifically even in p53-null SKOV-3 cells [32, 33]. We employed romidepsin to induce p21 protein and determined if CXCR2 affected romidepsin-induced p21 protein expression levels in p53-null cells. Although CXCR2 decreased p21 promoter activity in p53WT cells (Figure 3a), p53-null cells had no effects on p21 promoter activity in response to CXCR2 (Figure 6A). Romidepsin increased the p21 promoter activity and CXCR2 further potentiated romidepsin-induced p21 upregulation (Figure 6A). In addition, romidepsin increased p21 promoter activity in a dose-dependent manner (Figure 6B). Moreover, we assessed the effect of romidepsin on p21 promoter activity in deleted constructs of p21 promoter. Romidepsin had a similar response to all deleted constructs, including p53 RE deletion (Figure 6C), indicating no specific binding site for p53 RE to regulate p21 in p53-null cells.

### CXCR2-expressing cells are more resistant to the anti-tumor effect of romidepsin through p21 downregulation

**Figure 7:**
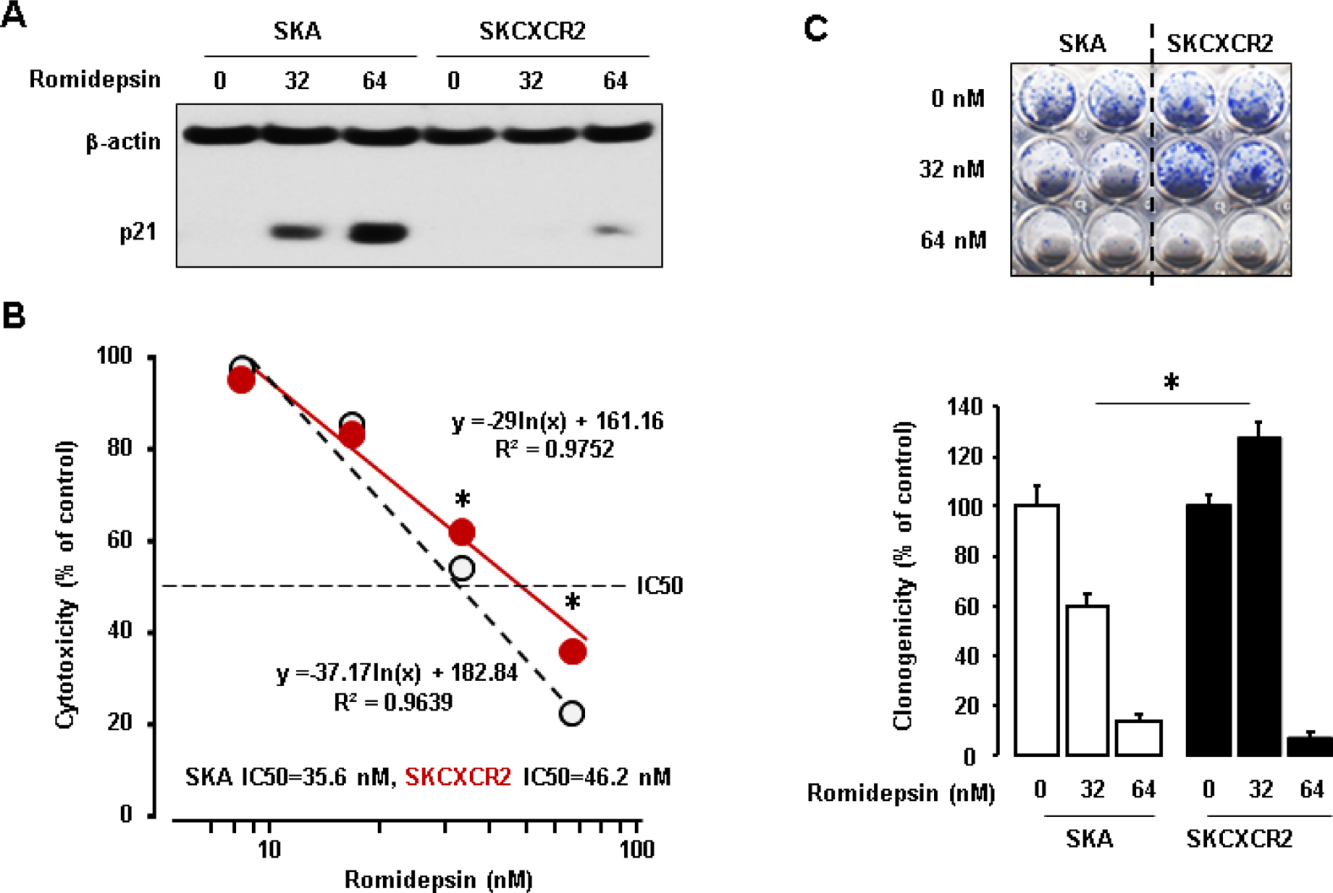
Resistant effects of CXCR2 on the anti-tumor potential of romidepsin in p53-null cells (**A**) Inhibitory effects of CXCR2 on romidepsin-induced p21 protein in SKA and SKCXCR2cells. Cells were treated with romidepsin (0, 32 and 64 nM) for 24 h. β-actin was detected as an internal loading control of cell lysates. A representative result is shown from duplicated experiments. (**B**) IC50 of romidepsin in SKA and SKCXCR2 cells. Cells were treated with 10–100 nM romidepsin for 48 h and then cell proliferation assay was performed. All data are shown as mean ± SE from triplicated experiments. ^*^indicates a significant increase (*p* ≤ 0.05) by Student’s *t*-test. (**C**) Clonogenic survival assay in SKA and SKCXCR2 after treatment of romidepsin (0, 32 and 64 nM) for 48 h. ^*^ (*p* ≤ 0.05) in each group by ANOVA and Tukey’s pairwise comparisons. All data are shown as mean ± SE from triplicated experiments. Each SE is located within circles.

We assessed differential effects of CXCR2 on romidepsin-induced p21 protein in SKA and SKCXCR2 cells. SKA cells increased p21 protein expression levels in response to romidepsin in a dose-dependent manner (Figure 7A). On the other hand, SKCXCR2 showed reduced romidepsin-induced p21 protein levels compared to SKA cells (Figure 7A). We compared IC50 of romidepsin in SKA and SKCXCR2 cells. SKCXCR2 cells had a higher IC50 of romidepsin (46.2 nM) compared to SKA cells (35.6 nM) (Figure 7B). Finally, we checked clonogenic survival assay after treatment with romidepsin in SKA and SKCXCR2 cells. SKCXCR2 cells had increased colony number compared to SKA cells at 32 nM of romidepsin below IC50 (Figure 7C). At 64 nM of romidepsin above IC50, both SKA and SKCXCR2 cells had similarly few colony numbers (Figure 7C).

### CXCR2 downregulates romidepsin-induced p21 protein expression through the Akt-Mdm2 axis in p53-independent manner in p53-null cells

**Figure 8:**
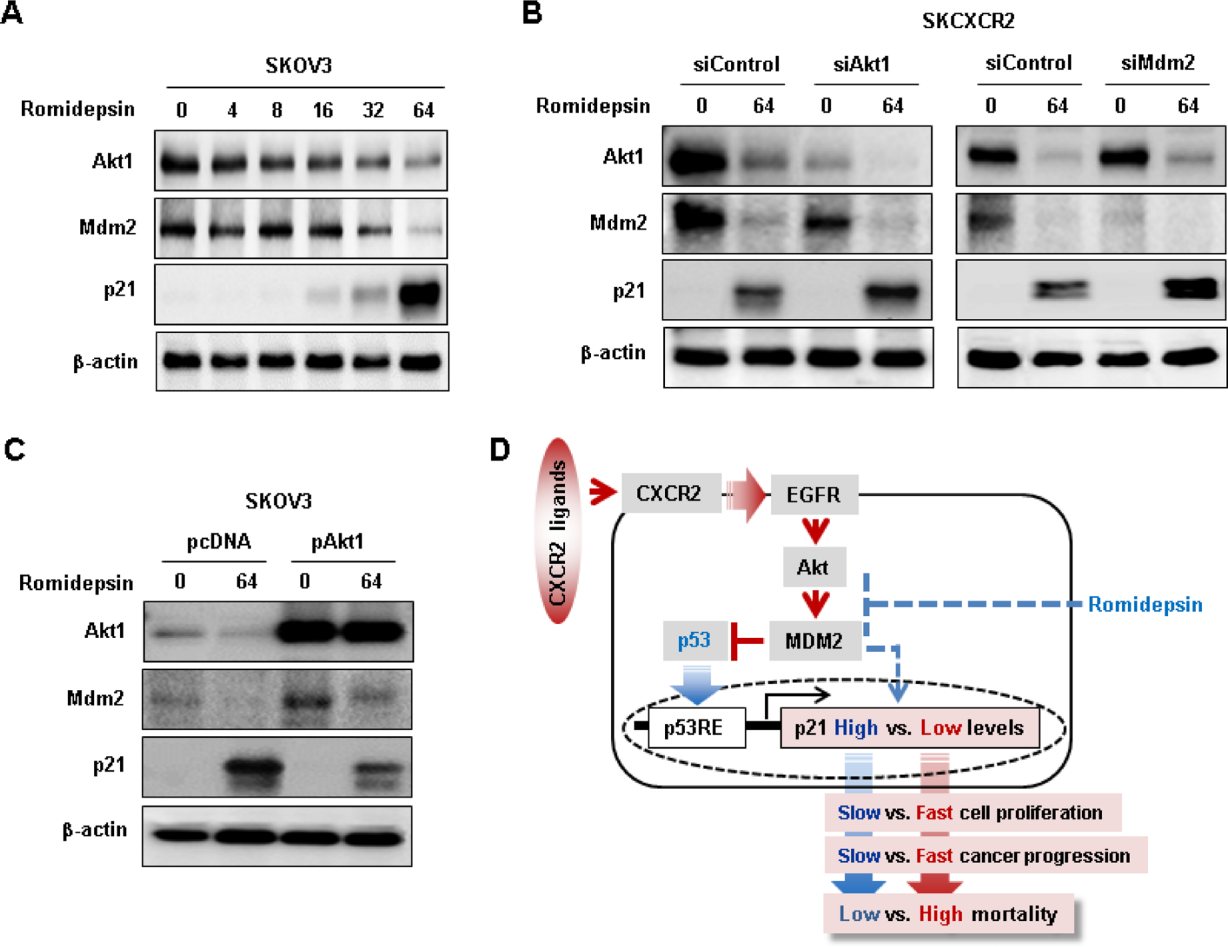
Negative effects of CXCR2 on romidepsin-induced p21 protein expression via Akt-Mdm2 axis in a p53-independent manner (**A**) Dose-dependent effects of romidepsin on Akt, Mdm2 and p21 protein expression in p53 null SKOV-3 cells. Cells was treated with 0, 4, 8, 16, 32 and 64 nM romidepsin for 24 h. (**B**) Effects of silencing Akt1 and MDM2 on romidepsin-induced p21 protein expression in SKCXCR2 cells. (**C**) Effects of overexpressed Akt1 on romidepsin-induced p21 protein expression in SKOV-3 cells. β-actin was detected as an internal loading control of cell lysates. Cells was treated with 64 nM romidepsin for 24 h. (**D**) Schematic representation of molecular mechanism of CXCR2-mediated Akt-Mdm2 axis on cell cycle inhibitor p21 regulation in p53-dependent and independent manner in ovarian cancer cells. A representative result is shown from duplicated experiments.

Since CXCR2 negatively regulated p21 through the Akt-Mdm2 axis in p53-dependent manner, we assessed if romidepsin utilized the Akt-Mdm2 axis to regulate p21 in p53-independent manner and if the CXCR2-activated Akt-Mdm2 axis could reduce romidepsin-induced p21 protein expression in p53-null cells. Romidepsin decreased Akt1 and Mdm2 protein levels followed by induced p21 protein expression levels in SKOV-3 cells in a dose-dependent manner (Figure 8A). Since SKCXCR2 cells expressed higher Akt and Mdm2 protein levels compared to SKA cells (Figures 3C and 5C), we then used SKCXCR2 cells to check if silencing Akt1 and Mdm2 could regulate romidepsin-induced p21 protein expression in a p53-independent manner. Knockdown of Akt1 decreased Mdm2 protein levels followed by enhanced romidepsin-induced p21 protein levels (Figure 8B). Although knockdown of Mdm2 had no effects on Akt protein levels, it increased romidepsin-induced p21 protein levels compared to control siRNA (Figure 8B). In addition, we overexpressed Akt1 into SKOV-3 cells to check if Akt-Mdm2 axis could reduce romidepsin-induced p21 protein expression in a p53-independent manner. Akt1 overexpression increased Mdm2 protein levels followed by reduction of romidepsin-induced p21 protein expression in p53-null SKOV-3 cells (Figure 8C).

## DISCUSSION

Our main finding is that CXCR2 negatively regulates p21 via Akt-mediated Mdm2 in p53-dependent and independent manner in ovarian cancer cell proliferation. Our previous study showed that CXCR2 transactivated EGFR, leading to Akt activation [19]. The Akt activation induces Mdm2, a key negative regulator of p53 [34]. Akt-mediated Mdm2 induction can increase p53 degradation which further inhibits cell cycle arrest protein p21 in a p53-dependent manner. The reduced p21 can enhance cell proliferation, reinforcing ovarian cancer progression followed by high mortality rate. Furthermore, CXCR2 inhibits HDACi-induced p21 in p53-null ovarian cancer cells via Akt-mediated Mdm2 in a p53-independent manner.

CXCR2-positive cells proliferated faster than CXCR2-negative cells, indicating that CXCR2 is a proliferative factor in ovarian cancer. Patients with highly CXCR2 expressed ovarian cancer had short survival compared to patients with low CXCR2 levels [16]. The p53WT cells are more responsive to CXCR2-mediated proliferation than p53-mutant and null cells, although all are somewhat responsive to CXCR2. Based on this fact, CXCR2 is likely inducing cell proliferation in both p53-dependent and independent manner in p53WT cells. On the other hand, p53-mutant and null cells are likely to only involve p53-independent CXCR2-induced proliferation. It is well established that Mdm2 interacts directly with p53, leading to loss of functional p53 roles [35]. Nutlin-3, a specific inhibitor of Mdm2 and stabilizing agent of p53WT [36], has anti-proliferative effects in p53WT cells, but not in p53-mutant and null cells, implying that nutlin-3 is p53-dependent. Furthermore, nutlin-3-stabilized p53 increased the expression levels of p21, a well-known direct p53 response gene. Despite similarity of nutilin-3 stabilized p53 levels between AA and ACXCR2 cells, CXCR2 reduced nutlin-3-induced p21, probably involving p53-independent mechanisms of CXCR2 in p21 regulation. In addition, CXCR2-mediated decrease of p21 promoter activity in p53WT cells, but not in p53-mutant and null cells, involves critical p53 RE in p21 promoter which indicates p53-dependent effects of CXCR2.

Many human cancers showed overexpressed Mdm2 [37, 38]. CXCR2 induced Mdm2 protein levels irrespective of p53 status in ovarian cancer cells. Some studies have shown that Akt stimulates the phosphorylation and translocation of Mdm2 into the nucleus, where it binds to p53 followed by loss of functional p53 roles [39–41]. Promoter, knockdown and overexpression studies have revealed that Akt-induced Mdm2 plays a critical role in CXCR2-mediated proliferation of ovarian cancer by inhibiting p53 function to induce p21. Therefore, CXCR2 could mediate Akt-induced Mdm2, resulting in inhibition of p53-dependent p21 upregulation in p53WT ovarian cancer, followed by increased cell proliferation and finally high mortality rate.

Almost all ovarian cancers (above 96%) are p53 mutant or null resulting in non-functional p53 activity, and p53WT ovarian cancers are rare [21]. In addition, p53-null status might be more metastatic than p53-mutant in ovarian cancer [42]. We selected p53-null SKOV-3 cells to clarify p53-independent effects of CXCR2 in cell proliferation. Because p53-null SKOV-3 cells did not express p21 protein at basal levels, romidepsin, a FDA approved HDAC class I inhibitor, was used as a p21 inducer in p53-independent manner as described previously [32, 33]. HDACs are important players in cancer development and progression [43]. HDACs were recruited to gene promoters to suppress the tumor suppressors and cell-cycle inhibitors [43, 44]. Treatment of cancer cells with HDACi results in apoptosis [45]. Cell cycle inhibitor p21 is one of the frequently induced gene by HDACi via releasing the repressor HDAC1 from its promoter [44–46]. Since romidepsin induces the activity of deleted p21 promoters even without p53 RE in p53-null cells, it is not likely to involve a specific region in p21 promoter to regulate p21 in a p53-independent manner. Furthermore, CXCR2-mediated downregulation of p21 promoter activity in p53WT cells could not be observed in p53-null cells. However, CXCR2 decreases romidepsin-induced p21 protein expression levels in p53-null cells. Based on the negative effects of CXCR2 on romidepsin-induced p21, IC50 of romidepsin and clonogenic colony formation, CXCR2 could be resistant to romidepsin treatment in ovarian cancer. The resistance to anti-tumor effect of romidepsin in CXCR2-positive cells might be due to the over-amplification of phosphorylated Akt. The Akt phosphorylation was implicated in deviating apoptotic signals in liver cancer [47], and the constitutive activation of Akt induced chemo- and radio-therapeutic resistance in small-cell lung cancers and gastric cancers [48, 49]. In human leukemia K562 cells, the HDAC6 inhibitor disrupts the antiapoptotic pathway of Akt [50]. We have confirmed that CXCR2 mediates Akt-induced Mdm2 in p53-null cells as observed in p53WT cells. Interestingly, romidepsin-induced p21 involves Akt-induced Mdm2 in a p53-independent manner by downregulating Akt and Mdm2 protein levels. CXCR2 is likely to activate Akt, leading to induction of Mdm2 which blocks p21. On the other hand, romidepsin is likely to downregulate Akt, leading to reduction of Mdm2 which potentiates p21.

In summary, CXCR2-driven ovarian cancer progression directly upregulates its own ligands such as CXCL1 and CXCL2 [19]. The released CXCL1 and CXCL2 binds to CXCR2, leading to activated EGFR [19]. The activated EGFR leads to phosphorylation of Akt, which directly upregulates Mdm2. The Akt-induced Mdm2 can suppress anti-tumor role of p53 followed by downregulation of cell cycle arrest protein p21, a p53 transcriptional target gene, which in turn can prompt cell proliferation, faster cancer progression and finally higher mortality rate (Figure 8D). HDACi like romidepsin is well known to induce p21 in p53-null ovarian cancer cells [32, 33], which can suppress the Akt-induced Mdm2 followed by slower cell proliferation and lower mortality rate (Figure 8D). CXCR2 enhances the Akt-induced Mdm2 which inhibits HDACi-induced p21 in a p53-independent manner, leading to faster cancer progression and higher mortality rate (Figure 8D). Conclusively, CXCR2 is a negative regulator of p21 via the Akt-induced Mdm2 in p53-dependent and independent manner in ovarian cancer.

## MATERIALS AND METHODS

### Reagents

Romidepsin (FK228) was obtained from Gloucester Pharmaceuticals (Cambridge, MA, USA). Pre-designed siRNAs for non-targeting Control, Akt1 and Mdm2 were purchased from Santa Cruz Biotechnology (Santa Cruz, CA, USA). Nutlin-3 was obtained from R&D Systems (Minneapolis, MN, USA). Antibodies were purchased as follows: Akt, p21 (waf1/cip1), Akt isoform and Akt phosphorylated form (pAkt, Ser473) from Cell Signaling Technology (Beverly, MA, USA) and p53, Mdm2, CXCR2, and β-actin from Santa Cruz Biotechnology (Santa Cruz, CA, USA). Fluorescein isothiocyanate-conjugated anti-CXCR2 (FAB331F, 1:1) was from R&D Systems (Minneapolis, MN, USA) and propidium iodide was from Sigma-Aldrich (St. Louis, MO, USA). PCR array for the cell cycle, SYBR^®^ Green Master Mix, RNeasy Mini Kit and RNase-free DNAse set came from SABiosciences in Qiagen (Frederick, MD, USA). Chemiluminescent detection kits were from GE Healthcare (Piscataway, NJ, USA). Penicillin G/streptomycin, Lipofectamine 2000 and all liquid culture media were acquired from Invitrogen (Grand Island, NY, USA). Mycoalert mycoplasma detection kit was from Lonza (Allendale, NJ, USA). G418-sulfate was purchased from Cayman Chemical (Ann Arbor, MI, USA). Finally, the Luciferase Reporter Assay System was obtained from Promega (Madison, WI, USA).

### Stable CXCR2 expressing cell lines and cell cultures

All cells were routinely tested for mycoplasma (Lonza, Allendale, NJ, USA) contamination according to the manufacturer’s protocol. Human ovarian cancer cell lines OVCAR-3 (p53-mutant) and SKOV-3 (p53-null) were purchased from the American Type Culture Collection (Manassas, VA, USA). A2780 (p53 wild-type) was kindly provided by Dr. Andrew Godwin (University of Kansas Cancer Center, Kansas City, KS, USA). CXCR2 expression vector was kindly provided by Dr. Ann Richmond (Vanderbilt University, Nashville, TN, USA) [51, 52]. Stable CXCR2 expressing cell lines were generated by transfecting CXCR2 or empty vectors into parental A2780, OVCAR-3 and SKOV-3 cells. Briefly, subconfluent cells were transfected with CXCR2 or empty vectors using lipofectamine 2000 and then treated with G418 to select drug-resistant clones. The cells were treated every 3 days with G418 until resistant-clones appeared. The expression of CXCR2 protein in the selected clones was confirmed by western blot and confocal imaging analysis. The CXCR2-positive cell lines were termed ACXCR2, OVCXCR2 and SKCXCR2 and the CXCR2-negative control cell lines, AA, OVA, and SKA, respectively. The cells were cultured in RPMI medium containing penicillin/streptomycin and 10% FBS at 37° C in a water-saturated atmosphere of 95% air and 5% CO_2_.

### Cell proliferation assays

Cell proliferation assays were performed using the cleavage of 3-(4,5-dimethylthiazol-2-yl)-2,5-diphenyltetrazolium bromide (MTT) to a colored product. After incubation in a 24-well plate, each well was washed twice with phosphate-buffered saline (PBS) and then MTT solution (1 mg/ml of phenol red-free media: PBS = 4:1) was added. The plates were incubated for 3 h with protection from light. The MTT solution was removed and 500 µl of isopropanol was added. The plates were placed on a shaker for 10 min at room temperature to thoroughly dissolve the MTT color product. Optical density was measured at 595 nm using a microplate reader (Bio-Rad, Hercules, CA, USA). Values were normalized to untreated controls.

### Confocal imaging analysis

Cells (5000 cells/250 µl media) were seeded on 8-chambered slides and cellular attachment was allowed overnight. The cells in the chamber slides were washed 3 times in PBS and fixed with 4% paraformaldehyde for 10 min at room temperature and blocked with 1% BSA in PBS for 30 min. The primary antibody was applied for 1 h at room temperature, and then washed 3 times with PBS for 30 min. The slides were then incubated with the second antibody conjugated with Alexa Fluor 594 or Alexa Fluor 488 (LI-COR Biotechnology, Lincoln, NE, USA) for 1 h at room temperature. Finally, the slides were washed 3 times with PBS, mounted with mounting medium containing DAPI (Vector laboratories, Burlingame CA, USA) and observed with a fluorescence microscope (Nikon A1R laser scanning confocal imaging).

### Flow cytometry

Cell cycle status were detected by flow cytometry using protocols described previously [53]. Cancer cells were seeded at equal densities and maintained in culture for 24 h. Briefly, 1 × 10^6^ cells of SKA and SKCXCR2 cells were harvested and washed twice with PBS. The cells were fixed on ice-cold 75% ethanol at 4° C for a minimum of 4 h and then washed twice with PBS. The cells were then treated with RNAse (Sigma-Aldrich) in a 37° C water bath for 20 minutes, then finally stained with propidium iodide [50 mg/ml in 0.1% (w/v) sodium citrate, 0.1% (v/v) Triton X-100] overnight. The cells were then analyzed by flow cytometry (FACStation, BD Biosciences) and the percentage of cells in G0/G1, S and G2/M phases were quantified utilizing FlowJo software (Tree Star Inc., Ashland, OR, USA). The assay was repeated in triplicate.

### Western blotting

Total whole-cell lysates were prepared, fractionated on SDS-polyacrylamide gels, and transferred to polyvinylidene difluoride membrane as described previously [54]. The total protein extract for each cell line was obtained by using a lysis buffer and equal amounts (30 µg per load) were analyzed by immunoblotting. Blocking of nonspecific proteins was performed by incubation of the membranes with 5% nonfat dry milk in Tris buffered saline Tween-20 (TBST) for 2 h at room temperature. Blots were incubated with primary antibodies at 1:1,000 dilution in blocking solution overnight at 4° C. The membranes were washed 3 times with TBST for 10 min, followed by incubation for 1 h with horseradish peroxidase conjugated secondary antibody (1:2,500 dilution) in 5% milk/TBST. The membranes were then rinsed 3 times with TBST for 10 min and the bands visualized by enhanced chemiluminescence. β-actin (sc-1616, Santa Cruz, CA, USA) was detected as an internal loading control of cell lysates.

### Transient transfection

Ovarian cancer cells were seeded to approximately 75% confluence in 6-well plates, were washed and transiently transfected with previously constructed HA epitope-tagged Akt1 [55], which was a kind gift from Dr. Joseph Testa (Fox Chase Cancer Center, Philadelphia, PA, USA). Briefly, cells were transfected with Akt1 or pcDNA3 expression vector (control) for 16 h at 37° C using Lipofectamine™ 2000 (Invitrogen Life Technologies, Grand Island, NY, USA), following manufacturer’s instructions. Transfection efficiencies were determined by western blot, and treated as outlined in Results.

### Luciferase assay

Ovarian cancer cells were seeded to approximately 75% confluence in 24-well plates, were washed and transiently transfected with p21 promoter subcloned into pGL2-basic luciferase reporter vector (a gift from Dr. Xiao-Fan Wang, Duke University Medical Center, Durham, NC, USA) for 16 h at 37° C using Lipofectamine™ 2000 (Invitrogen Life Technologies, Grand Island, NY, USA). Transfected cells were treated as outlined in Results and incubated for 16 h. Cells were then rinsed with ice-cold PBS and lysed with lysis buffer (Promega, Madison, WI), and finally cell lysates were used for determination of luciferase activity using a microplate luminometer. Luciferase activity, expressed as relative light units, was normalized to measured protein levels.

### Knockdown of Mdm2 and Akt1

Cells at approximately 75% confluency in 6-well plates were washed once with 1% FBS fresh media without additives and then transiently transfected with Control or Akt1 and Mdm2 specific siRNA (final concentration: 100 nM) for 72 h at 37° C using Lipofectamine solution. Transfected cells were confirmed knockdown of Akt1 and Mdm2 protein using western blots, and treated as outlined in Results according to various experiments.

### PCR array for cell cycle genes

Total RNAs were isolated from SKA and SKCXCR2 cells using RNeasy Mini Kit (Qiagen). Genomic DNA contamination was eliminated using the RNase-free DNAse set (Qiagen). cDNA was synthesized by RT reaction at 42° C for 15 min followed by 94° C for 5 min. A real-time PCR reaction was performed using the Human Cell Cycle RT^2^ Profiler PCR array (Qiagen) on a Bio-Rad CFX96 (Hercules, CA, USA) and the following two-step cycling program: 1 cycle at 95° C for 10 min, and 40 cycles at 95° C for 15 sec and at 60° C for 1 min. Data analysis was performed based on a Web-Based PCR Array Data Analysis (http://pcrdataanalysis.sabiosciences.com/pcr/arrayanalysis.php) provided by SABiosciences in Qiagen (Frederick, MD, USA).

### Clonogenic survival assay

Cells were grown in 6-well plates to 75% confluence, and then treated with vehicle or romidepsin for 48 h. After collecting cells with trypsin, 1000 cells/ml were then reseeded into 6-well plates and incubated with fresh media for an additional 7 days. Cells were fixed in methanol and then stained with (0.25% methylene blue in 50% methanol) for 30 min at room temperature. After washing with PBS and air-drying the plates, colonies were counted. The data were expressed as percent survival relative to the control.

### Statistical analysis

Data were analyzed by the paired Student’s *t*-test and one-way analysis of variance (ANOVA) as appropriate. If a statistical significance (*p ≤* 0.05) was determined by ANOVA, the data were further analyzed by Tukey’s pairwise comparisons to detect specific differences between treatment groups.

## Abbreviations

ANOVA: one-way analysis of variance
CDKN1A: cyclin dependent kinase inhibitor 1
HDACi: histone deacetylase inhibitor
MAPK: mitogen-activated protein kinase
Mdm2: murine double minute 2
MTT: 3-(4,5-dimethylthiazol-2-yl)-2,5-diphenyltetrazolium bromide
OC: ovarian cancer
PBS: phosphate buffered saline
PI3K: phosphoinositide 3-kinase
RE: responsive element
STAT3: signal transducer and activator of transcription 3
TBST: tris-buffer saline Tween 20
WT: wild-type
